# In Vivo Investigation of Cardioprotective Effects of *Melilotus officinalis* and *Melilotus albus* Aerial Parts Extracts for Potential Therapeutic Application

**DOI:** 10.3390/plants14172639

**Published:** 2025-08-25

**Authors:** Anca Toiu, Ana-Maria Vlase, Laurian Vlase, Tibor Casian, Alina Elena Pârvu, Ilioara Oniga

**Affiliations:** 1 Department of Pharmacognosy, Faculty of Pharmacy, “Iuliu Hațieganu” University of Medicine and Pharmacy, Ion Creangă Street 12, 400010 Cluj-Napoca, Romania; atoiu@umfcluj.ro (A.T.); ioniga@umfcluj.ro (I.O.); 2 Department of Pharmaceutical Botany, Faculty of Pharmacy, “Iuliu Haţieganu” University of Medicine and Pharmacy, 41 V. Babes St., 400012 Cluj-Napoca, Romania; gheldiu.ana@umfcluj.ro; 3 Department of Pharmaceutical Technology and Biopharmacy, Faculty of Pharmacy, “Iuliu Haţieganu” University of Medicine and Pharmacy, 41 V. Babes St., 400012 Cluj-Napoca, Romania; casian.tibor@umfcluj.ro; 4 Department of Morpho-Functional Sciences, Discipline of Pathophysiology, Faculty of Medicine, Iuliu Haţieganu University of Medicine and Pharmacy, 400012 Cluj-Napoca, Romania; parvualinaelena@umfcluj.ro

**Keywords:** *Melilotus officinalis*, *Melilotus albus*, cardioprotective agents, antioxidants, polyphenolic compounds

## Abstract

Globally, cardiovascular diseases represent a major cause of morbidity and mortality, despite the availability of preventive, diagnostic, and therapeutic measures in contemporary allopathic medicine. In accordance with their ethnomedical applications, herbal medicines may offer valuable options for the prevention, treatment, and management of cardiovascular disorders. Considering that cardioprotective effects are associated with antioxidant mechanisms, and that our knowledge of the antioxidant properties of polyphenolic compounds, as well as of the effects of *Melilotus* species on the heart, is limited, the present study aimed to evaluate the cardioprotective potential of hydroalcoholic extracts of *M. officinalis* and *M. albus* aerial parts. The extracts were evaluated for total phenolic content (TPC), total flavonoid content (TFC), and total coumarin content (TCC) by spectrophotometric methods and by LC-MS/MS. The effect of pretreatment with *Melilotus* sp. extracts on the isoprenaline-induced infarct-like lesion in rats was evaluated by ECG monitoring and the assessment of serum oxidative stress markers and serum cardiac injury markers. Various polyphenolic compounds were identified by LC-MS/MS in *M. officinalis* and *M. albus* aerial parts: catechin, syringic acid, protocatechuic acid, and vanillic acid. Gallic acid and chlorogenic acid were found only in *M. officinalis*. The extracts showed good in vivo antioxidant activity: *M. officinalis* and *M. albus* extracts induced a significant decrease in the levels of oxidative stress index (OSI) and total oxidant status (TOS), while pre-treatment with *M. albus* extract induced a significant reduction in nitric oxide production, and pretreatment with *M. officinalis* increased total thiols (SH) levels. In the same way, ECG and cardiac injury markers were also improved. These results show that *M. officinalis* and *M. albus* extracts may exert cardioprotective effects against myocardial ischemia by reducing oxidative stress.

## 1. Introduction

Certain cardiovascular diseases (CVDs), notably ischemic heart disease (IHD) and hypertension, are the primary cause of mortality in industrialized countries, and their prevalence is growing all over the world [1,2]. Myocardial infarction (MI) is an acute condition of myocardial necrosis which manifests as a disproportion between myocardial demands and coronary blood supply. Several biochemical mechanisms are implicated: an increased generation of reactive oxygen species (ROS); the oxidative deterioration of membrane proteins, carbohydrates, lipids, and DNA; inflammatory processes; and important changes in the structural, mechanical, electrical, and biochemical properties of the heart [1,3,4].

Many important phytochemicals from medicinal plants have been shown to alleviate the pathophysiology of acute myocardial infarction, improve conventional treatment, and offer better management of the disease with fewer adverse effects, as demonstrated in various studies [1,2,4]. Free radical scavengers and antioxidant compounds could help the myocardial cells recover from the induced programmed cell death; therefore, these compounds might be used as part of a preventive approach to overcome myocardial ischemia. Dietary administration of natural products has been proven to improve endothelial function and lipid metabolism, as well as to modulate oxidative stress pathways in experimental and clinical models of cardiovascular dysfunction [5]. Evidence for the role of polyphenolic compounds in the prevention of degenerative diseases due to their antioxidant activity is emerging. Several mechanisms have been proposed to explain the cardioprotective activity of polyphenols, such as activation of AMP-activated protein kinase, nitric oxide synthase, and sirtuin 1, improvement of endothelial cell function, inhibition of angiotensin-converting enzyme and phosphate diesterase, fibrinolytic activity, and anti-platelet aggregation activity. Furthermore, polyphenolic compounds lower blood pressure; possess anti-inflammatory potential; improve insulin resistance, ventricular health, and plasma lipid markers; and reduce atherosclerosis, which will lower the risk of cardiovascular diseases [6].

*Melilotus* species (Fabaceae family) are annual, biennial, or short-lived perennial herbs that occur frequently in spontaneous flora or are cultivated. In *Flora Europaea*, there are mentioned 16 species, while around 20–25 species are recognized worldwide [7,8]. In Romania there are five species: *M. dentatus*, *M. albus*, *M. arenaria*, *M. Altissimus*, and *M. officinalis* [9]. Research on bioactive compounds from *Melilotus* species has revealed the presence of coumarins, flavonoids, saponins, and steroids [10,11,12]. Used for centuries in traditional medicine for the treatment of arthritis, rheumatism, bronchitis, kidney stones, or palpitations, *M. officinalis* is the most studied species of this genus [8,13,14].

Several studies have shown the anti-inflammatory, antioxidant, and antiproliferative potential of *M. officinalis* aerial parts [15,16] for the prevention of skin aging, the reduction of fat deposition, or the promotion of tissue regeneration [17], while the European Medicines Agency [18] recommends the use of the vegetal product for minor venous circulatory disturbances (orally) or for minor inflammation of the skin and feelings of heaviness in the legs (topically). The European Pharmacopoeia mentions a minimum content 0.3% of coumarin, which relates to the dried plant material [19]. The phytochemical profile of *M. officinalis* consists mainly of cinnamic acid, melilotoside, coumarin, melilotin, scopoletin, umbelliferone, kaempferol, and quercetin glycosides [10,11,17].

*M. albus* is another *Melilotus* species that is relatively abundant among Romanian spontaneous Flora. It is a herbaceous biennial plant with white flowers and is considered very important for honey production. A few studies have noted the main active compounds as being the coumarins, flavonoids, and tannins from the aerial parts [20]; as well as their use as anticoagulants, to treat external ulcers in traditional medicine, or as antimicrobial and antibiofilm agents [12].

Because the content of bioactive compounds can vary due to their role in plant defence mechanisms (in response to various stressors) and the pedoclimatic conditions of the area of harvest, the use of a standardized extract is essential for determining therapeutic efficacy and limiting secondary reactions [21]. Taking into account the growing interest in the therapeutic potential of plant extracts and the well-documented cardioprotective activity of several major compounds (vanillic and protocatechuic acids) [22,23,24,25], but also the gap in the available literature concerning the evaluation of the cardioprotective properties of *Melilotus* species and the evaluation of bioactive compounds from plant material harvested from Romania, we propose to investigate the cardioprotective effects of several *M. officinalis* and *M. albus* samples from different areas.

Therefore, the aim of our research is to determine the phytochemical composition of *M. officinalis* and *M. albus* aerial parts collected from Romania and to evaluate their in vivo and in vitro antioxidant effects, as well as the cardioprotective potential of standardized extracts, in order to enhance the therapeutic value of herbal medicines and to justify their use based on scientific evidence. To the best of our knowledge, this is the first in vivo evaluation of the cardioprotective effects of *M. officinalis* and *M. Albus*; thus, our study is the first to demonstrate the valuable properties of these extracts in herbal remedies.

## 2. Materials and Methods

### 2.1. Chemicals and Reagents

In the LC-MS/MS phytochemical analysis, 25 polyphenolic standards were utilized. Analytical standards, including caftaric acid, chlorogenic acid, caffeic acid, p-coumaric acid, rutin, apigenin, kaempferol, luteolin, gentisic acid, myricetol, fisetin, (+)-catechin, (−)-epicatechin, quercetin, quercitrin, isoquercitrin, hyperoside, patuletin, protocatechuic acid, syringic acid, vanillic acid, and rosmarinic acid, with purity levels ranging from 78% to 98%, were purchased from Sigma-Aldrich Chemie GmbH, Schnelldorf, Germany. Gallic acid, with a purity of 98%, was procured from Merck, Darmstadt, Germany. Additionally, ferulic acid and sinapic acid, both with ≥98% purity, were obtained from Roth, Karlsruhe, Germany. The solvents employed for extraction and separation included methanol, ammonium acetate, and acetonitrile of HPLC analytical-grade, and petroleum ether, chloroform, hydrochloric acid, acetic acid, and potassium hydroxide of analytical-grade [26,27].

These solvents and Folin–Ciocâlteu reagent were purchased from Merck (Darmstadt, Germany). 6-hydroxy-2,5,7,8-tetramethylchroman-2-carboxylic acid (Trolox) and 2,2-diphenyl-1-picrylhydrazyl (DPPH) were purchased from Alfa-Aesar (Karlsruhe, Germany). Sodium carbonate, sodium acetate trihydrate, anhydrous aluminium chloride, coumarin, lead (II) acetate, 2,4,6-tri(2-pyridyl)-1,3,5-triazine (TPTZ) reagent, 2,2-diphenyl-1-picryl-hydrazyl-hydrate (DPPH) reagent, Trolox, and isoprenaline were obtained from Sigma-Aldrich (Schnelldorf, Germany). The commercial biochemistry kits for the pharmacological investigations (kit CK-MB-LQ. Anti CK-M. Immunoinh.; kit GOT/AST-LQ. IFCC. Enzymatic–UV; GPT/ALT-LQ. IFCC. Enzymatic–UV; kit UREA-LQ. Urease-GLDH. Kinetic; kit creatinine-J. J) were obtained from S.C. DG Diagnostics S.R.L. Cluj-Napoca [3,26].

### 2.2. Plant Material

*Melilotus officinalis* and *M. albus* aerial parts were harvested from wild populations during the flowering stage from different locations chosen through random selection. The voucher specimens were deposited in the Herbarium of the Pharmacognosy Department, University of Medicine and Pharmacy, Cluj-Napoca, as presented in Table 1. The plant material was authenticated by A.-M.V. in the Department of Pharmaceutical Botany. Each plant material was air-dried at room temperature, ground to a fine powder with an electric grinder, and used for extraction.

### 2.3. Preparation of Plant Extracts

The extraction of aerial parts from *M. officinalis* and *M. albus* was performed on a water bath at 60 °C for 50 min with a 1:10 ratio of sample (*m*/*v*) to 70% ethanol (*v*/*v*), followed by ultrasonic-assisted extraction for 50 min [28]. The obtained extracts were concentrated to dryness under reduced pressure at 40 °C. The dried extracts were stored at 4 °C until further analysis. The extracts yields were expressed in relation to the dry weight of the vegetal product (mg crude extract/g dry weight plant material). The polyphenolic compounds remained stable at 4 °C over the four-week storage period, with no significant decrease observed in their total content or antioxidant activity. It should be noted, however, that long-term stability may differ depending on the specific polyphenol class, the sample matrix, and exposure to environmental factors such as light or oxygen, which were controlled in the present study.

### 2.4. Quantitative Analyses of Total Bioactive Compounds

The total phenolic content (TPC) of the ethanol extracts from *M. officinalis* and *M. albus* aerial parts was determined by the Folin–Ciocâlteu method, as detailed previously [29]. The total phenolic content was expressed as mg gallic acid equivalents (GAEs)/g dry extract. The experiments were performed in triplicate.

The evaluation of the total flavonoid content (TFC) of the extracts from *M. officinalis* and *M. albus* was performed using a spectrophotometric method [30]. The flavonoids content was expressed as rutin equivalents (mg REs)/g dry extract. The experiments were performed in triplicate.

The total coumarin content (TCC) of the extracts from *M. officinalis* and *M. albus* was determined using the spectrophotometric method with a JASCO UV-VIS spectrophotometer. Briefly, this Borntrager reaction is based on the solubility of free coumarin derivatives in polar organic solvents and the solubility of their soluble alkali phenolates. The ionization of phenolic hydroxyls in the molecule by alkaline hydroxide causes a bathochromic deviation of 320 nm, which is proportional to the coumarin concentration. TCC was expressed as mg coumarin/g dry extract (mg CE/g dry extract). The experiments were performed in triplicate [31].

### 2.5. LC-MS/MS Analysis of Polyphenols Apparatus and Chromatographic Conditions

The ethanol extracts of *Melilotus* species were analyzed for their phytochemical composition using liquid chromatography–tandem mass spectrometry (LC-MS/MS). The instrument used was an Agilent 1100 HPLC Series system, equipped with an autosampler, binary gradient pump, degasser, and a column thermostat maintained at 48 °C, along with a UV detector. The mass spectrometric analysis was performed using an Agilent Ion Trap 1100 SL, featuring both electrospray ionization (ESI) and atmospheric pressure chemical ionization (APCI) sources. The system operated at a flow rate of 1 mL/min and an injection volume of 5 µL, using a Zorbax SB-C18 reverse-phase analytical column for compound separation.

Rosmarinic acid identification and quantification involved a mobile phase comprising acetonitrile and 1 mM ammonium acetate in water. A gradient elution began with 5% acetonitrile, increasing to 25% at 3.3 min (method a). The MS operated in negative ionization mode with ESI, targeting the fragmentation and isolation of the deprotonated rosmarinic acid molecule (*m*/*z* = 359).

For the remaining 24 polyphenolic compounds, two separate analytical methods were employed. The first method focused on 18 polyphenols, using a mobile phase of methanol and 0.1% acetic acid. The elution process utilized a binary gradient, starting with 5% methanol and reaching 42% at 35 min, followed by a 3 min isocratic elution. Detection was carried out using both UV and MS modes, with the UV detector set at 330 nm for polyphenolic acids and 370 nm for flavonoids and their aglycones. The MS conditions included a negative ionization mode with an electrospray ion source. The specific mass spectra signal for each compound was used for identification, while UV and MS assisted in quantification. A standard polyphenol solution was used to create a spectral library, with detection limits determined based on a signal-to-noise ratio greater than 3 (method b).

The second LC-MS method was applied to six polyphenols, with working conditions similar to those of method b, but with a different mobile phase gradient. In this case, the gradient commenced with 3% methanol, increased to 8% methanol at 3 min, and then to 20% methanol from 8.5 min to 10 min. Identification and quantification for these compounds were performed exclusively in the MS mode, under previously described conditions (method c).

Chromatographic data were collected and processed using DataAnalysis (v5.3) and ChemStation software (vB01.03) from Agilent Inc. The retention times of the compounds were established using reference standards and confirmed through mass spectrometry. Sample spiking with 10 µg/mL polyphenol solution ensured accuracy. Compound identification was based on comparing retention times and ESI-MS spectra with those of standards under identical conditions. Quantification of polyphenols in each extract employed the external standard method, with calibration curves linear in the range of 0.5–50 µg/mL (R^2^ > 0.999) [26].

### 2.6. The Evaluation of In Vitro Antioxidant Potential

The evaluation of the radical scavenging effect of the ethanol extracts from *M. officinalis* and *M. albus* aerial parts was achieved by DPPH and FRAP radical scavenging assays, and more detail can be found in [30]. The reference standard was Trolox. The antioxidant potential was expressed as IC_50_ (μg TE/mL) for the DPPH assay and as Trolox equivalents (TEs)/100 mL of extract for the FRAP method. All determinations were performed in triplicate.

### 2.7. The Evaluation of In Vivo Cardioprotective Effects

#### 2.7.1. Experimental Protocol

The in vivo experiments were performed on adult male Wistar albino rats (weigh 200–250 g) from the “Iuliu Hațieganu” University of Medicine and Pharmacy Animal Facility. Prior to and during the experiments, animals had ad libitum access to water and free access to standard pellets-based diet (Cantacuzino Institute, Bucharest, Romania) and were housed in proper conditions (12 h night/day cycle, temperatures of 21–22 °C, and humidity of 50–55%). All treatments that involved animals were in accordance with EU Directive 2010/63/EU on the protection of animals used for scientific purposes and guidelines for Animal Welfare. The experimental design of animal study was approved by the Institutional Animal Ethical Committee (IAEC) of the “Iuliu Hațieganu” University of Medicine and Pharmacy Cluj-Napoca and by the National Sanitary Veterinary and Food Safety Agency (no. 382/94).

The experimental groups were organized by simple random allocation, and the treatments were administered each day at the same hour in order to minimize potential confounding factors.

#### 2.7.2. Experimental Myocardial Ischemia

For the evaluation of the cardioprotective potential of *Melilotus officinalis* and *M. albus* aerial parts, the animals were divided randomly into five groups (n = 5). Group 1—negative control (CONTROL); Group 2—isoprenaline (ISO); Group 3—*Melilotus officinalis* extract (MO2); Group 4—*Melilotus albus* extract (MA2); and Group 5—positive control coumarin (CUM). The animals received the following: (1) CONTROL—0.9% saline solution by gavage 1 mL/day for 7 days; (2) ISO—0.9% saline solution by gavage 1 mL/day for 7 days; (3) MO2—*Melilotus officinalis* extract 10% in distilled water (*m*/*V*) by gavage 1 mL/day for 7 days; (4) MA2—*Melilotus albus* extract 10% in distilled water (*m*/*V*) by gavage 1 mL/day for 7 days; (5) CUM—coumarin solution by gavage 25 mg/kg BW, for 7 days. The animals from groups (2)–(5) received isoprenaline subcutaneously 150 mg/kg BW on days 8 and 9 in order to induce MI [3,32]. The electrocardiogram (ECG) was recorded on days 2, 7, and 10. On day 10, after ECG registration, blood samples from animals were collected under general anaesthesia (induced by 70 mg ketamine/kg BW and 10 mg xylasine/kg BW) for the estimation of cardiac markers and oxidative stress markers [33]. The samples were stored at −80 °C until further analysis, and cardiac markers GOT, GPT, and CK-MB were evaluated using commercial kits.

#### 2.7.3. Electrocardiography Results

In several cardiac abnormalities, such as myocardial infarction or myocardial ischemia, changes in the normal ECG pattern may appear. Therefore, in order to evaluate the effects of the extracts on the heart’s electrical activity, the ECGs were recorded. The overnight-fasted rats were anesthetized with 60 mg ketamine/kg BW and 8 mg xylazine /kg BW (*intraperitoneal administration*). At 15 min after anaesthesia, animals were placed in the supine position on a board, electrodes were bound on the paw pads of each rat, and ECG was recorded from the limb lead at position II (right forelimb to left hind limb) with a Biopac MP150 system. The ECG apparatus was calibrated at 1 mV/1 cm with a speed of 50 mm/s. Analysis of ECG waves was conducted as previously described [34]. Briefly, heart rate (beats/min), RR intervals (ms), QT interval (ms), and ST segment changes (mV) were calculated. The corrected QT interval (QTc), which is used to rectify the influence of the heart rate on the QT interval, was also calculated according to Bazett’s formula [35].

#### 2.7.4. The Evaluation of In Vivo Antioxidant Properties

The total oxidative status (TOS) of the serum was measured as previously reported, using a colorimetric method with hydrogen peroxide (H_2_O_2_) as standard. In the presence of different oxidant species, a ferrous ion is oxidated into a ferric ion, and the absorbance is measured using a spectrophotometer. The results were presented in µmol H_2_O_2_ equivalents/L [36]. The total antioxidant capacity (TAC) was determined as previously reported, using Trolox as standard [37]. The oxidative stress index (OSI) represents an indicator of the degree of oxidative stress and is calculated as the ratio of the TOS (μmol H_2_O_2_ Equiv/L) to the TAC (mmol Trolox Equiv/L) [38,39]. Malondialdehyde (MDA) was evaluated as a lipid peroxidation marker using thiobarbituric acid, as previously reported, with 1,1,3,3-tetraethoxypropane as standard, and was expressed as μM/L [40]. Total thiols (SH) were estimated using Ellman’s reagent, as previously mentioned, with glutathione (GSH) as standard, and were expressed as μM GSH/L [41]. Initially, serum proteins were removed [42]; then, the serum NO concentration was determined using the Griess reaction and a sodium nitrite-based curve and expressed as nitrite μmol/L [43]. All spectrophotometric measurements were performed using a Jasco V-530 UV-Vis spectrophotometer (Jasco International Co., Ltd., Tokyo, Japan).

### 2.8. Multivariate Analysis

Multivariate analysis of data was accomplished using principal component analysis (PCA). PCA was implemented to assess the effect of species type and harvesting region on the quality of extracts, particularly the phytochemical composition. Variables, including TFC, TPC, TCC, DPPH, and FRAP, were scaled to unit variance, and biplots were generated for the interpretation of the model.

**Figure 1:**
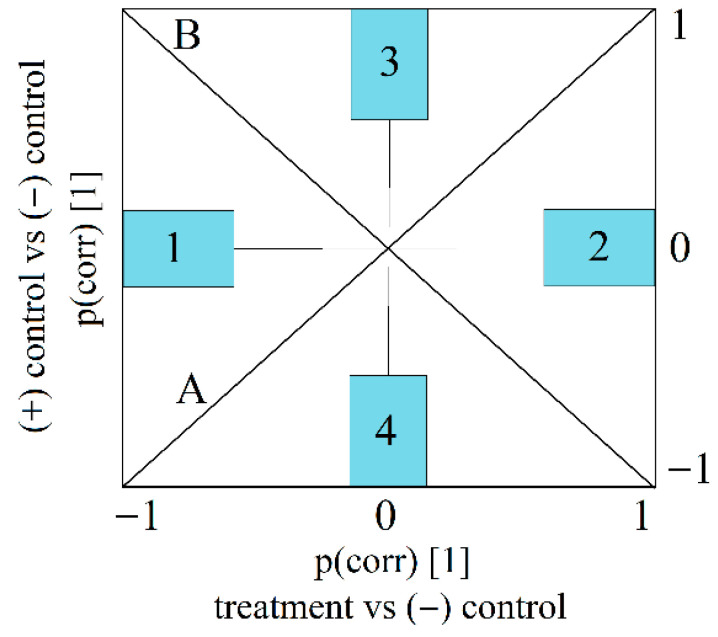
The interpretation of SUS plots. Note: shared effects: Diagonal A—variables influenced in the same direction. Diagonal B—variables influenced in opposite direction with respect to the (−) control. Unique effects—unique decrease (Region 1) and unique increase (Region 2) induced by “treatment”; unique increase (Region 3) and unique decrease (Region 4) induced by (+) control.

Orthogonal projections to latent structures-based discriminant analysis (OPLS-DA) were applied to investigate the treatment-induced differences in the expression profile of the oxidative stress parameters and cardiac markers. The X dataset (variables) and the Y dataset (class membership dummy variables) were scaled to unit variance before model development. Model performance was evaluated by considering the fraction of explained variability (R2X, R2Y) and the predictive capacity (Q2). Separate models were developed between an applied treatment and the negative control. The p(corr) vectors of two OPLS-DA models were represented (SUS plots) to highlight the shared and unique contribution of the applied treatments with respect to the investigated variables. The vertical axis of SUS figures plots the correlation vector of the model that discriminates positive (ISO) and negative control, while the horizontal axis refers to the comparison of another treatment and negative control (Figure 1).

Shared effects are identified through variables displayed on diagonal A, where both treatments lead to an increase (positive) or a decrease (negative) in the variables. Opposite effects can be identified through variables placed on diagonal B, while unique effects are associated with regions 1–2–3–4. In case of unique effects, the variable has a high absolute value on only one axis [44,45].

### 2.9. Statistical Analysis

The analyses were performed in triplicate, and the obtained results were expressed as means ± standard deviation (SD). Data were compared using one-way ANOVA and post hoc Bonferroni–Holm test. Pearson’s correlation coefficient (*r*) was used to evaluate relationships between parameters of the same group. The statistical significance of differences between data was evaluated by SPSS 20.0 for Windows (SPSS Inc., Chicago, IL, USA). The level of significance was established at *p* < 0.05.

## 3. Results and Discussion

### 3.1. Quantitative Analyses of Total Bioactive Compounds

The quantitative determinations of the main active compounds from the *M. officinalis* and *M. albus* aerial parts extracts are presented (Table 2). *M. officinalis* from the Brasov county (MO2) extract contains higher quantities of active constituens than one from Cluj county (MO1). Regarding other extracts, there are small differences between the *Melilotus albus* extract from Alba county (MA2) and the one from Cluj county (MA1). The amount of TPC from MO2 is higher than that from MA2 (138.157 mg GAE/g versus 127.279 mg GAE/g), and the same trend was observed regarding the total coumarins (47.691 mg CE/g, and, respectively, 37.331 mg CE/g), while for total flavonoids, it was reversed (78.201 mg RE/g versus 86.105 mg RE/g).

Our results are in accordance with those published by other researchers. For instance, Ayadi et al. determined the total polyphenols in ethanol extracts from *M. officinalis* aerial parts from Morocco, and their quantity increased from 2.14% at the vegetative stage to 2.37% at the flowering stage, while between some ecotypes, the level was found to be between 2.01% and 2.69% [46]. In another research from 2020, Szymanski et al. found 2.101% to 2.438% total polyphenols and 0.748% to 0.975% total flavonoids in *M. officinalis* water extracts, whilst *M. alba* aerial parts were found to contain a higher amount of polyphenols (2.7–3.5%) and flavonoids (0.8–1.1%) [47]. Another study on *M. indicus* showed that aqueous extracts contain a higher quantity of total polyphenols (144.382 µg GAE/ mg extract) and flavonoids (25.65 µg QE/ mg) than ethanol extracts (120.067 µg GAE/mg extract; flavonoids: 26.375 µg QE/mg) [48].

The differences between the results could be explained by different extraction techniques, the solvent used, other variable extraction parameters, or the manner of reporting the results. Thus, the research on *M. officinalis* flowers from Iran presents values between 48.21 and62.4 mg QUE/g for flavonoids, while the total phenolic content has values between 24.32 and 38.08 mg GAE/g [49].

Previous research on the coumarin content of *Melilotus* sp. aerial parts grown in South Australia showed that it can range from 0.06 to 0.75%, and the variations are determined by extraction procedures and solvents, as well as the plant species. *M. officinalis* contains 0.332% coumarins (between 0.09 and 0.61%) and *M. albus* 0.523% (between 0.06 and 1.3%) [50]. The authors mention the importance of evaluating the coumarin content in each sample since there are significant differences between them, and the biological activities might be influenced.

The variation in polyphenol quantities in different plant samples could be determined by genetic or ecological factors or the status of secondary metabolites in various growing locations, as previously shown [51].

Due to the results obtained from the evaluation of total bioactive compounds, for further research, the plant materials MO2 (*M. officinalis* from Brasov County) and MA2 (*M. alba* from Alba County) were chosen for additional investigations.

### 3.2. LC-MS/MS Analysis of Polyphenols

The phytochemical analysis of *M. officinalis* and *M. albus* extracts was performed using a targeted LC-MS/MS technique for the identification and quantification of several poyphenolic compounds, namely, flavonoids and phenolic acids, and the results are presented in Table 3.

Chlorogenic, ferulic, protocatechuic, vanillic, syringic, and rosmarinic acids, as well as catechin, were found in higher quantities in the *M. officinalis* extract, with vanillic acid (57.37 ± 2.36 mg/100 g extract) and protocatechuic acid (40.64 ± 3.51) being the main compounds. Chlorogenic and gallic acids were determined only in *M. officinalis* extracts, as well as caftaric acid, which was found in small quantities (<LOQ). *p*-Coumaric acid was identified in both extracts, but the content is below the limit of detection.

The major compound of both investigated extracts, vanillic acid, is a monohydroxybenzoic acid found in numerous medicinal plants. Several recent studies showed the antioxidant properties, as well as its anti-inflammatory, antidiabetes, and antiallergic effects of this compound, which are related to the reduction in lymphocytes, neutrophils, eosinophils, and macrophage levels, the reduction in TNF-α, IgE, IL-4, and IL-5 levels, and the increasing IFN-γ levels in serum [52].

Another recent study by Magiera et al. demonstrated the antioxidant and anti-inflammatory properties for vanillic acid in human neutrophils and plasma, as well as in vitro on non-cellular models [53].

Protocatechuic acid is another phenolic compound identified in both *Melilotus* sp. extracts, with high quantity in *M. officinalis* aerial parts. Protocatechuic acid has recently been the subject of several studies, which revealed its important therapeutic effects; it has neuroprotective, anticancer, analgesic, anti-inflammatory, antihyperglycemic, and antioxidant properties, which are useful both in the prevention and treatment of several diseases [54]. Other studies found that it has chemopreventive potential due to its inhibition of in vitro chemical carcinogenesis, and it exerts pro-apoptotic and anti-proliferative effects in different tissues [55].

The results obtained in this study are in line with those of other authors on some polyphenols from *Melilotus* species. Higher quantities of *o*- and *p*-coumaric acids were determined in dried flowers compared with fresh ones. The content in *o*-coumaric acid was 1.00 mg/g in *M. officinalis* and 0.34 mg/g in *M. albus* dried samples, while in the fresh ones, it was below LOQ. *p*-Coumaric acid content was 0.16 mg/g in *M. albus* dry flowers and 0.13 mg/g in fresh ones; while in *M. officinalis*, it was 0.12 mg/g and 0.07 mg/g, respectively [56].

The important qualitative and quantitative differences between *M. officinalis* and *M. albus* aerial parts allow for a better correlation between the chemical composition and pharmacological properties of the extracts and justify the use of standardized extracts for the in vivo evaluation of plant extracts’ biological properties.

Compared to other polyphenol-rich foods, the plants investigated in this study demonstrate a distinct phytochemical profile, characterized not only by high total polyphenol content but also by unique combinations of specific bioactive compounds, including phenolic acids, flavonoids, and coumarins not commonly found in widely consumed sources. This compositional uniqueness may underlie the observed in vivo cardioprotective and antioxidant effects, which have not been previously reported for *M. officinalis* and *M. albus* extracts. Furthermore, the potential synergistic interactions between these compounds could confer enhanced biological activity, positioning these plants as promising candidates for the development of novel functional foods or nutraceuticals with targeted health benefits.

### 3.3. In Vitro Antioxidant Properties

The results of the evaluation of antiradical properties of *M. officinalis* and *M. albus* extracts are presented (Table 4). The ethanol extracts of *M. officinalis* aerial parts with higher total polyphenolic content had a better antioxidant effect in the DPPH test (IC_50_ = 92.19 μg TE/mL and 118.03 μg TE/mL, respectively). The same tendency was observed for *M. albus* extracts (IC_50_ = 52.19 μg TE/mL and 73.25 μg TE/mL, respectively). Another study found values of DPPH scavenging activity 9.0 μg/mL for hexane extract, 35.6 μg/mL for 96% ethanol extract, and 30.2 μg/mL for 50% ethanol extract of *M. officinalis* [57]. The results cannot be directly compared with those of the present study since they were expressed as the % inhibition. Other authors showed that in *M. alba*, the higher content of polyphenols (3.01%) correlated with the lower antioxidant activity of water extracts (IC_50_ = 5.31 mg/mL) when compared with *M. officinalis*, with 2.43% polyphenols and IC_50_ = 4.35 mg/mL [47]. The authors revealed variability in the antioxidant activity, which depends on the extract concentration as well as the solvent used.

Another recent study by Sowa et al. on the antioxidant properties of *Melilotus* sp. found a higher antioxidant effect in leaf extracts when compared with flower extracts in DPPH and FRAP tests: for *M. albus* flower extract, 49.18 µmol TE/g (DPPH test) and 53.77 µmol TE/g (FRAP test); for *M. officinalis* flower extract, 73.26 µmol TE/g and 82.72 µmol TE/g, respectively [56]. A comparison with the present study is not possible since these differences might be due to the distinct extraction method and plant organ evaluated, as well as the manner in which the results are presented and the differences in methodology.

### 3.4. Evaluation of In Vivo Cardioprotective Activity

The cardioprotective effects of *M. officinalis* and *M. albus* were determined in vivo using a rat model of isoprenaline-induced acute myocardial infarction. The therapeutic efficacy was evaluated by analyzing oxidative stress markers (OSI, TAC, MDA, NO_x_, SH, and TOS), along with the biochemical indicators characteristic of myocardial injury (GOT, GPT, and CK-MB) (Table 5).

Myocardial infarction induction led to significant increases in serum GOT and GPT levels (*p* < 0.01) and CK-MB levels (*p* < 0.001) when compared to the control group. This was accompanied by a pronounced oxidative imbalance, evidenced by elevated TOS, OSI (*p* < 0.001), and NO_x_ levels (*p* < 0.05), along with a significant reduction in SH levels (*p* < 0.01).

Relative to the isoprenaline group, pretreatment with the MA2 extract significantly reduced TOS and OSI levels (*p* < 0.001). CUM produced a small increase (*p* < 0.05), whereas MO2 showed no statistically significant effect (*p* > 0.05). CUM pretreatment led to a highly significant increase in TAC levels (*p* < 0.01), while MO2 and MA2 elicited moderate yet significant improvements on this parameter (*p* < 0.05).

Regarding lipid peroxidation, MO2 and MA2 markedly lowered MDA levels (*p* < 0.001), with CUM having a milder but still significant effect (*p* < 0.01). A significant reduction in NO_x_ concentrations was observed with MO2 (*p* < 0.05) and MA2 (*p* < 0.01), while CUM did not influence this parameter. MA2 extract significantly increased SH levels, with the most pronounced effect observed for MO2 and CUM (*p* < 0.001), whilst MA2 produced a moderate but significant response (*p* < 0.05).

Taking into account the myocardial injury markers, MA2 significantly reduced GOT (*p* < 0.01), GPT levels (*p* < 0.001), and CK-MB (*p* < 0.001); MO2 extract and CUM did not produce statistically significant changes in GOT and GPT parameters (*p* > 0.05), whilst CK-MB was significantly reduced by MO2 and CUM (*p* < 0.001) (Table 6).

ECG recording from days 1 and 7 showed no significant changes in all groups. The ECG patterns of normal- and experimental-group rats on day 10 are shown in Table 7. Isoprenaline administration resulted in notable ECG alterations, including a reduced RR interval and increased heart rate (HR), QT, and corrected QT (QTc) intervals. PR, QRS, and ST were insignificantly changed by isoprenaline-induced infarct-like lesion. RR was increased by the pretreatment with MO2 (*p* < 0.01), MA2 (*p* < 0.5), and CUM (*p* < 0.001). HR was significantly reduced by MO2, MA2, and CUM (*p* < 0.001); QT was moderately yet significantly reduced by MO2 (*p* < 0.05) and CUM (*p* < 0.001); and QTc was decreased significantly only by CUM (*p* < 0.001).

When comparing the two extracts, both MO2 and MA2 exhibited significant antioxidant and cardioprotective effects, with some variation in their activity profiles. MO2 and MA2 significantly reduced oxidative stress markers such as MDA, TOS, and OSI and enhanced antioxidant defenses (TAC and SH). However, MA2 demonstrated slightly greater efficacy in decreasing OSI and reducing NO levels—a change not observed with CUM (*p* > 0.05).

With respect to cardiac injury markers, both MO2 and MA2 significantly decreased GOT, GPT, and CK-MB levels, though MA2 exerted a slightly stronger effect on all markers.

Isoprenaline (ISO) is a synthetic catecholamine with toxic effect on the myocardium. The generation of ROS via the auto-oxidation of catecholamines, increased lipolysis, and the peroxidation of endogenous lipids are considered the main causative factors of isoprenaline-induced cardiac damage. The isoprenaline-induced MI increases cAMP formation due to an enhancement in adenylate cyclase activity; therefore, it leads to a higher lipid accumulation in the myocardium [1,3]. The rat model of ISO-induced MI is a well-accepted standardized model by which to determine the effects of several synthetic and natural cardioprotective agents, as well as the cardiac dysfunctions [1,4]. The biochemical, electrocardiographic, and histological modifications closely imitate that observed in acute MI and is considered a relevant model. Several natural products, extracts, and single- or polyherbal formulations are employed in traditional medicines for the treatment of ischemic heart diseases, showing major benefits due to their antioxidant and/or anti-inflammatory effects [4].

In this isoprenaline-induced myocardial infarction model, Trolox served as the positive control due to its well-documented antioxidant properties [58]. Trolox administration resulted in significant improvements in oxidative stress parameters, including reductions in NO levels (*p* < 0.01), as well as TOS, OSI, and MDA levels (*p* < 0.05). These effects were accompanied by significant increases in TAC and SH levels (*p* < 0.01). Furthermore, Trolox exhibited cardioprotective activity, as evidenced by significantly reduced serum CK-MB levels (*p* < 0.001) and GOT and GPT levels (*p* < 0.05). These findings confirm the validity of the experimental model and establish a reference point for evaluating the cardioprotective potential of MO2 and MA2 extracts.

Several studies demonstrated the cardioprotective effects of vanillic acid—a phenolic compound identified in both *Melilotus* sp. extracts. There are several mechanisms involved in cardioprotection, such as the antioxidant effect by reducing oxidative stress and the progression of heart disease, the improving of cardiac function by increasing the NO levels and FRAP value and improving left ventricular function, and the anti-inflammatory effect via the reduction in pro-inflammatory cytokines and cardiac enzymes levels, such as GOT and CK-MB [22,23].

Another polyphenolic compound identified in both *Melilotus* sp. extracts is protocatechuic acid. Its cardioprotective effects have been proven in several studies, along with its anti-inflammatory (by reducing the levels of pro-inflammatory cytokines), anti-apoptotic (through the increase in the expression of anti-apoptotic proteins like B-cell lymphoma 2 and the decrease in the expression of pro-apoptotic proteins like Bax and caspase-3; therefore, it prevents cardiomyocyte loss and fibrosis), and antioxidant properties (by means of reducing the levels of oxidative stress markers like malondialdehyde while increasing the levels of antioxidant enzymes like glutathione, superoxide dismutase, and catalase in cardiac tissue) [24,25].

Another polyphenol present in *M. officinalis* and *M. albus* extracts is syringic acid. A number of studies have reported its potential as a therapeutic candidate for preventing and treating cardiovascular diseases, especially those associated with cardiac hypertrophy, fibrosis, and myocardial infarction. Syringic acid has been shown to mitigate isoproterenol-induced cardiac hypertrophy and fibrosis in vivo and in vitro, has demonstrated protective effects by reducing infarct size and improving erythrocyte morphology, and it also possesses antioxidant and anti-inflammatory properties. The downregulation of Ereg, the inhibition of apoptosis in cardiomyocytes, the modulation of inflammatory pathways like NF-κB by scavenging free radicals and reducing oxidative stress, and the reduction of myocardial ischemia reperfusion injury by activating the PI3K/Akt/GSK-3β signaling pathway are several prospective mechanisms of syringic acid [59,60,61].

### 3.5. Multivariate Analysis

PCA was implemented to assess the effect of the harvest region and species type on the quality of the extracts, especially on the cardioprotective and in vivo antioxidant effects. Variables including DPPH, TFC, TPC, TCC, and FRAP were scaled to unit variance, and biplots were generated for the interpretation of the model.

PART 1

**Figure 2:**
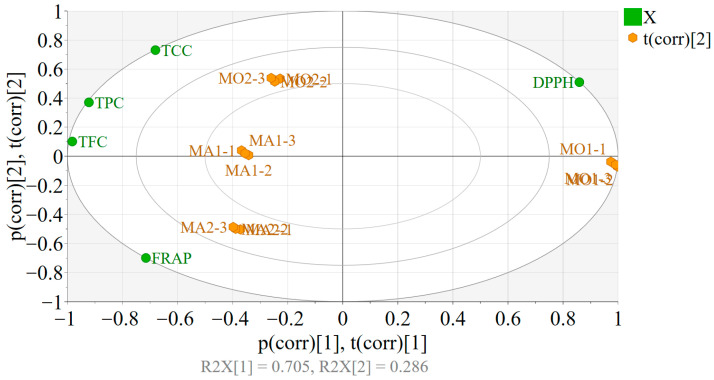
Biplot of PCA model.

The two PCs fitted on the obtained data explained 99.1% of the variability, enabling the identification of differences between the extracts (Figure 2). The positioning of the observations (extracts) in the score plot suggested a larger variability between the two *Melilotus officinalis* samples compared to the *Melilotus albus*-based extracts. Overall, the replicates performed within each extract were highly reproducible. The MO1 samples showed larger DPPH and lower TCC-TPC-TFC values compared to MO2, whereas both *M. officinalis* showed lower FRAP than *M. albus*. The differences between the two *M. albus* extracts were reserved mostly to the FRAP, TCC, and DPPH variables. MA2 exhibited higher FRAP and lower TCC and DPPH than MA1. In a similar way, MA1 had higher FRAP and lower TCC and DPPH than MO2.

PART 2

**Figure 3:**
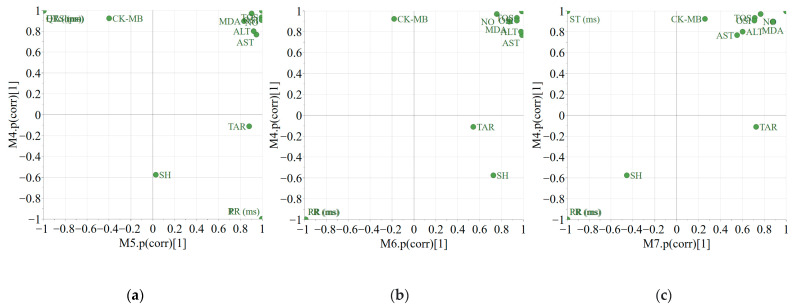
SUS plots generated by representing the p(corr) vector of OPLS-DA models developed between the control and other treatments (M4 Control vs. ISO; (**a**) M5—Control vs. ISO/CUM; (**b**) M6—Control vs. MO2 /ISO; (**c**) M7—Control vs. MA2/ISO).

SUS plots offer an easy visualization of the shared and unique effects exhibited by two different treatments compared to a control treatment. The shared effects can be identified by looking at the variables situated in the upper-right and lower-left corners. Thus, the administration of ISO (M4), CUM/ISO (M5), MO2/ISO (M6), or MA2/ISO (M7) led to an increase in the variables placed in the upper-right corner. The treatment with MA2/ISO had a lesser effect on GOT and GPT compared to MO2/ISO and CUM/ISO. The PR and RR variables, placed in the lower-left corner, suggested that the treatments with ISO, MO2/ISO, or MA2/ISO produced a negative effect, leading to a decrease (Figure 3b,c). From Figure 3a, the opposite effect was identified for the co-administration of CUM/ISO as it offered an increase in PR and RR.

A positive effect on CK-MB was identified only for the administration of ISO, whereas CUM/ISO (M5), MO2/ISO (M6), and MA2/ISO (M7) did not have an influence. Regarding the QRS-QTC-HR variable group, the administration of MO2/ISO, MA2/ISO, and ISO offered a positive effect compared to the control treatment, whereas the administration of CUM/ISO produced a negative effect.

Apart from MA2/ISO, exposure to ISO, CUM/ISO, and MO2/ISO produced an increase in ST.

Compared to the control treatment, exposure to ISO was not significant for TAC, whereas the use of MO2/ISO, MA2/ISO, and CUM/ISO offered—in the order presented—an increasing positive effect.

The positioning of the SH variable suggested a limited effect coming from the applied treatments.

## 4. Conclusions

The findings of this study suggest that hydroalcoholic extracts from the aerial parts of *Melilotus officinalis* and *M. albus* possess notable cardioprotective potential. Pretreatment with these extracts mitigated isoprenaline-induced myocardial injury in rats, likely through antioxidant mechanisms, as evidenced by improvements in oxidative stress and cardiac injury markers. The presence of bioactive polyphenolic compounds, such as syringic acid, vanillic acid, protocatechuic acid, and catechin further, supports their therapeutic relevance. Notably, *M. officinalis* exhibited unique constituents like gallic acid and chlorogenic acid, which may contribute to its distinct biological effects. Both *Melilotus* species exhibited high total polyphenol content, with substantial levels of flavonoids and coumarins. Overall, these findings suggest that *Melilotus* sp. extracts hold promise as natural agents for the prevention or management of cardiovascular diseases linked to oxidative stress.

Continued investigation is warranted to isolate and characterize the bioactive constituents and clarify their underlying mechanisms of action. The present findings highlight the therapeutic relevance of both *Melilotus* sp. extracts and introduce novel evidence regarding their cardioprotective effects which had not previously been reported.

The potential applications of these findings extend to the formulation of functional foods, nutraceutical products, and standardized plant-based preparations designed to support cardiovascular health and potentially mitigate the risk of related disorders. Such applications could be integrated into preventive healthcare strategies, particularly for populations with elevated cardiovascular risk. While the observed bioactivities suggest that dietary supplementation with *M. officinalis* and *M. albus* may be a viable approach, the current evidence remains preliminary. Before it can be responsibly recommended, further validation through well-designed, large-scale clinical trials is necessary to confirm the efficacy, optimal dosage, and safety profile of the extracts, as well as their standardization in bioactive compounds. In addition, considerations regarding bioavailability, potential interactions with conventional medications, and long-term effects are essential for the responsible translation of these results into public health recommendations.

## Figures and Tables

**Table 1:** *M. officinalis* and *M. albus* samples, voucher specimens, and GPS coordinates of harvesting places.

Sample	Plant Species	Voucher Specimen	Harvesting Place (GPS Coordinates)
MO1	*Melilotus officinalis*	MO-5	Cluj County (Lat. 46°83′64.37″ N, Long. 23°63′19.88′′ E)
MO2	*Melilotus officinalis*	MO-8	Brasov County (Lat. 45°39′48.57″ N, Long. 25°30′21.13″ E)
MA1	*Melilotus albus*	MA-4	Cluj County (Lat. 46°48′22.88″ N, Long. 23°35′17.77″ E)
MA2	*Melilotus albus*	MA-7	Alba County (Lat. 46°18′22.51″ N, Long. 23°07′51.55″ E)

**Table 2:** The quantitative analysis of bioactive compounds from *Melilotus* sp. extracts.

Extract	TPC (mg GAE/g de)	TFC (mg RE/g de)	TCC (mg CE/g de)
MO1	86.516 ± 0.98	34.752 ± 0.57	18.473 ± 0.63
MO2	138.157 ± 3.91	78.201 ± 1.63	47.691 ± 0.95
MA1	121.995 ± 3.05	77.278 ± 2.02	26.877 ± 0.55
MA2	127.279 ± 3.48	86.105 ± 1.66	37.331 ± 0.58

Note: de—dried extract; TPC—total phenolic content; GAE—gallic acid derivatives; TFC—total flavonoid content; RE—rutin equivalent; TCC—total coumarin content; CE—coumarin equivalent. Each value represents the mean of three determinations ± SD.

**Table 3:** Polyphenolic composition of MO2 and MA2 ethanol extracts by LC-MS (mg/100 g extract).

[M − H]^−^ *m*/*z*	Polyphenolic Compound	LOQ μg/mL	Main Daughter Ions	R_T_ ^a,b,c^ ± SD (min)	Polyphenol Content MO2	Polyphenol Content MA2
311	Caftaric acid ^b^	0.2	148.6, 178.6	3.34 ^b^ ± 0.05	<LOQ	nd
353	Chlorogenic acid ^b^	0.2	178.7, 190.7	5.6 ^b^ ± 0.05	4.33 ± 0.25	nd
163	*p*-Coumaric acid ^b^	0.2	118.7	9.18 ^b^ ± 0.08	<LOQ	<LOQ
193	Ferulic acid ^b^	0.2	133.7, 148.7, 177.6	12.8 ^b^ ± 0.10	1.28 ± 0.04	0.69 ± 0.02
169	Gallic acid ^c^	0.2	169 *	1.5 ^c^ ± 0.09	12.06 ± 0.37	nd
153	Protocatechuic acid ^c^	0.2	153 *	2.8 ^c^ ± 0.15	40.64 ± 3.51	8.01 ± 0.85
167	Vanillic acid ^c^	0.2	167 *	6.7 ^c^ ± 0.17	57.37 ± 2.36	23.48 ± 1.91
197	Syringic acid ^c^	0.2	197 *	8.4 ^c^ ± 0.11	11.55 ± 1.74	2.6 ± 0.05
289	Catechin ^c^	0.2	289 *	6.00 ^c^ ± 0.03	2.02 ± 0.05	1.84 ± 0.06
359	Rosmarinic acid ^a^	0.2	160.6, 178.6, 196.7	2.2 ^a^ ± 0.18	1.51 ± 0.09	<LOQ

Note: <LOQ—below limit of quantification; nd—not determined; ^a^—method a for HPLC analysis; ^b^—method b for HPLC analysis; ^c^—method c for HPLC analysis. Each value represents the mean of three determinations ± SD; *—MS1 (SIM) analysis mode.

**Table 4:** In vitro antioxidant effect (MO1, MO2, MA1, MA2).

Extract	DPPH Method IC_50_ (μg TE /mL)	FRAP Method μM TE/100 mL
**MO1**	118.036 ± 2.84	193.55 ± 3.24
**MO2**	92.192 ± 2.63	279.35 ± 4.96
**MA1**	73.256 ± 2.04	489.89 ± 8.71
**MA2**	52.193 ± 1.45	702.92 ± 9.65

Note: TE—Trolox equivalent; IC_50_—half-maximal inhibitory concentration. Each value represents the mean of three replicates ± SD.

**Table 5:** Markers of in vivo oxidative stress for MO2 and MA2 extracts.

Group	TAC (mM TE/L)	TOS (μM H_2_O_2_ E/L)	OSI	NO_x_ (μM/L)	MDA (μM/L)	SH (μM/L)
CONTROL	1.088 ± 0.001	18.008 ** ± 2.182	16.538 ** ± 1.996	27.564 *** ± 2.234	2.530 *** ± 0.334	444.400 *** ± 53.785
ISO	1.088 ± 0.001	32.456 ± 3.975	29.086 ± 4.559	41.820 ± 2.147	3.902 ± 0.417	377.200 ± 58.178
MO2/ISO	1.090 ^#^ ± 0.002	33.084 ± 6.415	31.247 ± 6.412	33.347 ^#^ ± 5.277	3.243 ^###^ ± 0.042	561.000 ^###^ ± 106.151
MA2/ISO	1.090 ^#^ ± 0.002	24.746 ^#^ ± 3.853	21.857 ^#^± 4.158	31.407 ^##^ ± 0.730	3.447 ^###^ ± 0.163	404.750 ^#^ ± 16.029
CUM/ISO	1.091 ^##^ ± 0.001	40.310 ± 1.210	36.944 ± 1.108	41.242 ± 4.833	3.528 ^##^ ± 0.430	444.800 ^###^ ± 83.125

Note: * *p* < 0.05; ** *p* < 0.01; *** *p* < 0.001 versus CONTROL; ^#^ *p* < 0.05; ^##^ *p* < 0.01; ^###^ *p* < 0.001 versus ISO. TOS—total oxidative status; OSI—oxidative stress index; TAC—total antioxidant capacity; NO_x_—total nitrites and nitrates; MDA—malondialdehyde; SH—total thiols; TE—Trolox equivalents; U/L—units/liter. Each experimental group included five animals. Treatment groups and the doses administered: CONTROL—0.9% saline solution, 1 mL/rat/day, no myocardial induction of infarction; ISO—distilled water, 1 mL/rat/day + isoprenaline (150 mg/kg) s.c. on days 8 and 9 for myocardial infarction induction; MO2—*Melilotus officinalis* extract 1 mL/day by gavage, for 7 days + isoprenaline; MA2—*Melilotus albus* extract 1 mL/day by gavage, for 7 days + isoprenaline; CUM—coumarin solution by gavage 25 mg/kg, for 7 days + isoprenaline. Values are expressed as mean ± SD (n = 5).

**Table 6:** Cardiac markers for in vivo cardioprotective evaluation of MO2 and MA2 extracts.

Group	GOT (U/L)	GPT (U/L)	CK-MB (U/L)
CONTROL	34.400 *** ± 4.506	37.200 *** ± 4.266	8.000 *** ± 1.732
ISO	84.800 ± 32.568	87.600 ± 28.962	17.600 ± 3.209
MO2/ISO	76.667 ± 1.528	78.667 ± 11.060	7.333 ^###^ ± 3.055
MA2/ISO	55.000 ^##^ ± 29.597	60.667 ^###^ ± 29.771	8.667 ^###^ ± 0.577
CUM/ISO	77.800 ± 12.518	85.800 ± 17.584	6.800 ^###^ ± 1.304

Note: * *p* < 0.05; ** *p* < 0.01; *** *p* < 0.001 versus CONTROL; ^#^ *p* < 0.05; ^##^ *p* < 0.01; ^###^ *p* < 0.001 versus ISO, U/L—units/liter, GOT—glutamate oxaloacetate transaminase, GPT—glutamate pyruvate transaminase, CK-MB—creatine kinase–MB isoenzyme. Each experimental group included five animals. Treatment groups and the doses administered: CONTROL—0.9% saline solution, 1 mL/rat/day, no myocardial induction of infarction; ISO—distilled water, 1 mL/rat/day + isoprenaline (150 mg/kg) s.c. on days 8 and 9 for myocardial infarction induction; MO2—*Melilotus officinalis* extract 1 mL/day by gavage, for 7 days + isoprenaline; MA2—*Melilotus albus* extract 1 mL/day by gavage, for 7 days + isoprenaline; CUM—coumarin solution by gavage 25 mg/kg, for 7 days + isoprenaline. Values are expressed as mean ± SD (n = 5).

**Table 7:** ECG values determined for in vivo cardioprotective potential of MO2 and MA2 extracts.

Group	RR (ms)	HR (bpm)	PR (ms)	QRS (ms)	QT (ms)	QTc (ms)	ST (mV)
CONTROL	0.231 *** ± 0.019	261.600 *** ± 21.338	0.049 ± 0.006	0.037 ± 0.006	0.066 *** ± 0.003	0.137 *** ± 0.007	0.030 ± 0.007
ISO	0.144 ± 0.016	420.200 ± 45.008	0.045 ± 0.006	0.039 ± 0.006	0.103 ± 0.006	0.273 ± 0.013	0.040 ± 0.008
MO2/ISO	0.192 ^##^ ± 0.061	337.000 ^###^ ± 96.374	0.043 ± 0.005	0.128 ± 0.197	0.093 ^#^ ± 0.014	0.221 ± 0.056	0.031 ± 0.008
MA2/ISO	0.157 ^#^ ± 0.028	393.250 ^#^ ± 71.672	0.041 ± 0.009	0.041 ± 0.005	0.101 ± 0.011	0.256 ± 0.015	0.027 ± 0.009
CUM/ISO	0.266 ^###^ ± 0.017	226.600 ^###^ ± 14.415	0.049 ± 0.004	0.035 ± 0.002	0.068 ^###^ ± 0.005	0.133 ^###^ ± 0.013	0.033 ± 0.004

Note: * *p* < 0.05; ** *p* < 0.01; *** *p* < 0.001 versus CONTROL; ^#^ *p* < 0.05; ^##^ *p* < 0.01; ^###^ *p* < 0.001 versus ISO. Each experimental group included five animals. Treatment groups and the doses administered: CONTROL—0.9% saline solution, 1 mL/rat/day, no myocardial induction of infarction; ISO—distilled water, 1 mL/rat/day + isoprenaline (150 mg/kg) s.c. on days 8 and 9 for myocardial infarction induction; MO2—*Melilotus officinalis* extract 1 mL/day by gavage, for 7 days + isoprenaline; MA2—*Melilotus albus* extract 1 mL/day by gavage, for 7 days + isoprenaline; CUM—coumarin solution by gavage 25 mg/kg, for 7 days + isoprenaline. Values are expressed as mean ± SD (n = 5).

## Data Availability

The raw data supporting the conclusions of this study are available upon request.

## Abbreviations

ECG	Electrocardiogram
HPLC-MS	High-Performance Liquid Chromatography–Mass Spectrometry
DPPH	2,2-Diphenyl-1-picrylhydrazyl
FRAP	Ferric Reducing Antioxidant Power
IC_50_	Half Maximal Inhibitory Concentration
TOS	Total Oxidant Status
OSI	Oxidative Stress Index
TAC	Total Antioxidant Capacity
MDA	Malondialdehyde
NO	Nitric Oxide
SH	Total Thiols
GOT	Glutamate Oxaloacetate Transaminase
GPT	Glutamate Pyruvate Transaminase
CK-MB	Creatine Kinase–MB Isoenzyme
CVD	Cardiovascular Disease
IHD	Ischemic Heart Disease
LDL	Low-Density Lipoprotein
CRP	C-Reactive Protein
ROS	Reactive Oxygen Species
LDH	Lactate Dehydrogenase
ATP	Adenosine Triphosphate
NAD	Nicotinamide Adenine Dinucleotide
SOD	Superoxide Dismutase
MO	*Melilotus officinalis*
MAS	*Melilotus albus*
TPC	Total Phenolic Content
GAE	Gallic Acid Equivalents
TFC	Total Flavonoids Content
RE	Rutoside Equivalents
QE	Quercetin Equivalents
CUM	Coumarin
TCC	Total Coumarin Content
CE	Coumarin Equivalents
TPTZ	2,4,6-Tripyridyl-s-triazine
Trolox	6-hydroxy-2,5,7,8-tetramethylchroman-2-carboxylic acid
APCI	Atmospheric Pressure Chemical Ionization
BW	Body Weight
ISO	Isoprenaline
MAPK	Mitogen-Activated Protein Kinase
NF-KB	Nuclear Factor Kappa B
TE	Trolox Equivalents
INFL	Inflammation
CAT	Catalase
GSH	Glutathione
GPx	Glutathione Peroxidase
OPLS-DA	Orthogonal Projections to Latent Structures based Discriminant Analysis
PCA	Principal Component Analysis
TNF-alfa	Tumor Necrosis Factor-alpha
IL-6	Interleukin-6
MI	Myocardial Infarction
LOQ	Limit Of Quantification
